# Rhythmotheraphy, or a Discussion of the Physiologic Basis and Therapeutic Potency of Mechano-Vital Vibration

**Published:** 1906-11

**Authors:** 


### Books and Magazines.

RHYTHMOTHERAPHY, OR A DISCUSSION OF THE PHYSIOLOGIC BASIS AND THERAPEUTIC POTENCY OF MECHANO-VITAL VIBRATION; to which is added a Dictionary of Diseases, with Detailed Suggestions as to the Technic of Vibratory Therapeutics, with Illustrations. By Samuel S. Wallian, A. M., M. D., Chicago. Ouellette Press, 1906. Price, $1.50; postage, 10 cents.

Mechanical vibration, as applied to therapeutic resources, has practically compelled a respectable recognition. This handsome volume, from the Ouellette Press, Chicago, is the most recent and in many respects the most satisfactory contribution to the literature of the subject yet produced. Wasting neither time nor space in preliminaries, introductions or prefaces, Dr. Wallian plunges at once into the logical and physiological aspects of his subject, and the result is as might be expected, of practical and helpful value to every practitioner. Such works are usually padded by compilation, pointless citations and theory-spinning. This volume is singularly free from any effort in any of these directions, and the busy practitioner, whether using a vibrator or not, will find plenty of valuable suggestions within its covers. The work is handsomely but not effusively illustrated, without in a single instance advertising the wares or appliances of any manufacturer.

